# Dynamics of university students' experiences with COVID-19 in the first year of pandemic in Russia: dataset

**DOI:** 10.3389/fpsyg.2023.1286036

**Published:** 2023-11-15

**Authors:** Natalia Antonova, Ksenia Eritsyan, Nina Usacheva, Larisa Tsvetkova

**Affiliations:** ^1^Centre for Interdisciplinary Basic Research, HSE University, Saint-Petersburg, Russia; ^2^Department of Sociology, HSE University, Saint-Petersburg, Russia; ^3^Department of Psychology, Herzen State Pedagogical University of Russia, Saint-Petersburg, Russia

**Keywords:** COVID-19, preventive behavior, perceived threat, mental health, misconceptions, university students, Russia, dataset

## 1 Introduction

The worldwide spread of COVID-19 in early 2020 affected all major spheres of social, including higher education (Pokhrel and Chhetri, 2021). Throughout the world, university students have been affected by campus closures and unplanned rapid shifts to online learning as a precautionary measure against the spread of the disease (Bozkurt et al., 2020) coupled with the introduction of non-pharmaceutical interventions such as social distancing, social isolation, and mask wearing.

Fast-changing contexts such as during the pandemic are useful to study how external factors and stressful events lead to changes in behavior, psychological state and response to proposed preventive measures. It is also important to investigate the stability of behavior patterns over time.

There is robust evidence for temporal shifts in adherence to protective behaviors during the course of pandemic. However, the data regarding how exactly behavior is being changed are inconsistent. The most common dynamic is the gradual decrease of preventive behavior over time, as people adjust to the effect of the disease after the initial period (Petherick et al., 2021; Reicher and Drury, 2021). A possible explanation for the decline in adherence is “pandemic fatigue”—the idea of behavioral tiredness brought on by following COVID limitations (Reicher and Drury, 2021). In the COVID-19 pandemic context, psychological fatigue would result in a decreased motivation and/or capacity to adhere to sometimes difficult and unpleasant protective measures over an extended period of time (Michie et al., 2020). However, the findings of a longitudinal study conducted in the UK revealed the inverse pattern: there was an increase in reported health protective behaviors between the beginning and middle of pandemic (Schneider et al., 2021).

These differences can also have been heterogeneous across types of protective behaviors. Petherick et al. (2021) based on the data gathered in 14 countries have shown a linear rise in adherence to mask wearing and a U-shaped physical distancing pattern over time. The authors refer to this distinction to the difference in costs between the behaviors: the physical distance is a high-cost and sensitizing behavior while mask wearing could be considered low-cost and habituating.

Among factors associated with adherence to protective health behaviors and overall experience of life in the middle of the pandemic, researchers focus on the range of cognitive and mental health variables. One neglected cognitive factor is awareness of cases in people's social networks. People make judgments about the frequency or likelihood of events based on how available information is to them (Schwarz et al., 1991). As the pandemic spread, a person becomes more accustomed to the disease's unfavorable effects, such as its progression with complications or fatalities from COVID. When one knows someone who has suffered severe consequences of COVID-19, perception of the COVID-19 threat may increase, while knowing someone who has suffered mild consequences could have no effect or have the opposite effect (Anson and Eritsyan, 2023). Being exposed to information that someone has severely suffered or died from COVID-19 could also serve as an emotional experience which is a dramatic relief (Prochaska and Velicer, 1997) and directly influence preventive behavior.

In contrast, trust is one of the most widely discussed country-level moderators of adherence to preventive behavior in the literature (Devine et al., 2020). Preventive behavior is influenced by trust in authorities, including political and medical ones (Dryhurst et al., 2020). This association is also true for the youth group (Nivette et al., 2021). Trust serves as a major between risk perceptions and the willingness to implement preventive behavior: as interpersonal and/or institutional trust increases, the risk perception has a greater impact on precautionary behavior assumed (Diotaiuti et al., 2021).

Conspiracy theories and incorrect beliefs were found to be highly prevalent during COVID-19 pandemics; these are associated with a reduction in protective behaviors (Roozenbeek et al., 2020; Somma et al., 2022). However, despite being positively correlated, different forms of conspiracy beliefs have distinct behavioral implications. For example, conspiracy beliefs that describe the pandemic as a hoax were less strongly associated with preventive behavior, whereas conspiracy beliefs that sinister forces created the virus on purpose were associated with an increased level of preventive behavior (Imhoff and Lamberty, 2020).

Social norms also play an important role in predicting different health behaviors (Bilancini et al., 2020), including the ones that matter across COVID context. Perceived social norms are a reliable predictor for people's social distancing behaviors such as abstaining from personal contact (Schumpe et al., 2022) while little information is available regarding the evolution and predictive power of these social norms over time.

There is evidence that symptoms of distress-related psychopathology (anxiety; depression) can be exacerbated by COVID-19 and pandemic-related stressors in both the general population (Santabárbara et al., 2021; Filindassi et al., 2022; Robinson et al., 2022) and children and young people, who are the least medically vulnerable populations (Blendermann et al., 2023; Madigan et al., 2023; Sun et al., 2023). It was found that mental health characteristics were significantly associated with social preventive behaviors: higher levels of depressed symptoms are negatively related with recommended protective measures (Ding et al., 2020; Liu et al., 2020; Stickley et al., 2020). Despite much study on anxiety/depression as a factor in health-promoting activities in the setting of the COVID-19 epidemic, the strength and scope of the relationships between symptoms of anxiety/depression and protective behavior is unclear, especially in young adults.

Overall the COVID-19 pandemic has had a significant impact on many facets of health and society and has revealed the complex associations between behavioral, cognitive, and mental health variables. The present dataset offers secondary analytic opportunities to explore changes in the rates of those variables from the beginning of the first wave to the second wave of the pandemic. It also provides an opportunity to check the strength of relationships between different behavioral, cognitive, and mental health variables across those two data points. Based on a sample of Russian students it provides an opportunity to test analytical hypotheses on a non-WEIRD sample (Henrich et al., 2010) which is important for a nuanced understanding on the level of generalizability of research findings.

## 2 Materials and methods

### 2.1 Study design

This research used a repeated cross-sectional design. Data was collected during two waves of a routine monitoring survey of students' lifestyle and wellbeing at one of the largest universities in Saint-Petersburg (Russia). All research protocols and procedures were reviewed and approved by the Ethics Committee of Herzen State Pedagogical University of Russia (IRB00011060, record #19).

A convenience sample of university students was recruited into the routine monitoring online survey of students' life conducted by university officials. The section of items regarding the pandemic presented in the dataset was integrated in this monitoring study. Students were notified about the study via university based social media and communication platforms and encouraged to participate on the basis of anonymity. No compensation for the participation in the study was provided. All the data collection was implemented online. The participants provided consent before a data collection, indicating that they had read and understood the conditions of participation and the aims of the study. Among those who clicked on the invitation to participate in the study and read informed consent 97.2% agreed to participate.

There were two waves of data collection in this study. First wave took place in May–June 2020, second wave in September-November 2020. Since some of the students graduated and left university while others enrolled in the university between those two events, we excluded the graduate student's responses from the first wave dataset and first-year students from the second wave dataset. This enabled the study to include only those who were university students during both waves of data collection.

The dataset is presented in the two forms. The vertical format of dataset combined from the both waves consist of 1,790 observations (*n* = 1,017 in first wave and *n* = 773 in second wave) from a predominately female student population (86.4% of total sample, 86.7% in first wave, 85.9% in second wave). The average age of study participants was 20.77 years (*SD* = 2.78). In the first wave of data collection the average age of study participants was 20.79 years (*SD* = 3.00), in the second wave-−20.73 years (*SD* = 2.47).

Since the participation was anonymous to keep opportunity to merge data about same participants from different waves the participants were encouraged to create their personal code. Data from small subset of respondents (*N* = 197) which were identified as participated in the both waves was merged in the horizontal dataset which provides opportunity to analyze the-individual changes.

#### 2.1.1 Epidemiological context

The spread of COVID-19 in each country has its own unique characteristics (e.g., Feng et al., 2020; World Health Organization, 2020a). Thus, in the early stages of the pandemic, there was no consensus regarding the future trajectory of COVID-19 (Scudellari, 2020; Brüssow, 2021). In Russia by the middle of April 2020, COVID-19 cases had been detected in all its federal regions. St. Petersburg is one of the largest cities in the country and which has one of the most intensive spread of COVID-19 epidemic (Akimkin et al., 2021). The first round of data collection occurred at the peak of the first wave of COVID-19 pandemic in Russia (03/30/2020 to 08/30/2020) with an incidence of 51.31 cases per 100,000 people (Akimkin et al., 2022). In the study site a set of restrictive measures combined with the introduction of “non-working days” (03/30/2020 to 05/11/2020) was implemented. As a result university campuses were closed, and all learning, teaching, and assessment was moved online (Watermeyer et al., 2021).

The second round of data collection was at the beginning of the second wave of COVID-19 (08/31/2020 to 05/09/2021) after a decline in infections during the summer months. The number of daily confirmed cases exceeded the peak of the first wave and the number of daily confirmed deaths also was rapidly increasing (Mathieu et al., 2020). At that moment in Russia universities worked in a blended format but no lockdown was introduced.

Although the first vaccine against COVID-19 was registered in Russia in August 2020, mass vaccination was not available in Russia until December 4, 2020. That is why non-medical means of protection such as individual health-protective behavior were the only possible means to mitigate the spread of COVID-19 during both rounds of data collection.

### 2.2 Measures

The measures for the data collection were chosen considering the need to decrease the burden on respondents and increase the data quality. The construction of all variables was in line with the tools recommended by WHO for COVID-19 behavioral studies (World Health Organization, 2020b). The codebook is presented in Supplementary material.

#### 2.2.1 COVID-19 preventive behaviors

Behavior was measured based on the 7 days' recall period (SteelFisher et al., 2012; World Health Organization, 2020b). The five COVID-19 preventive behaviors were measured: social distancing, social isolation (staying at home), wearing masks in public places, wearing gloves, and washing hands more often than usual. The extent of respondents' engagement in these practices was evaluated with the question “To what extent do the following statements describe your behavior in the last week?” The response options were rated on a 5-point Likert scale, from never (1) to always (5).

#### 2.2.2 Perception of current COVID-19 stage

To measure how the respondents evaluate the current epidemic situation in the region they are located in, the following question was used: “In your opinion, the most difficult period of the epidemic of Coronavirus in the area where you are located…” with response options: “is in the past,” “is now,” or “is ahead”.

#### 2.2.3 Trust in official institutions

Two questions were used to evaluate the level of trust in the official medical recommendations and governmental support: “How much do you trust the official medical recommendations on the COVID prevention?” and “How much do you trust the government in how they care about the citizens during the COVID-19 pandemic?” [5 point Likert scale, from absolutely distrust (1) to absolutely trust (5); α = 0.431].

Additionally, a scale was developed and constructed for measuring the extent of respondents' endorsement of COVID-related misconceptions and conspiracy ideas (4 items, α = 0.562). The scale was focused on the respondents' evaluation of COVID-19 risks, related preventive measures, and conspiracy ideas behind them. The latter included external forces, for example, the role of the government in the spread of the pandemic and the lack of trust in the official medical recommendations related to COVID-19. The respondents were asked to evaluate how much they agree or disagree with such statements as “There is no difference between Coronavirus and ordinary influenza” or “In order to avoid infection, it is enough to strengthen the immune system, for example, by using folk remedies,” “Coronavirus is generally underestimated as a threat” (REV), “In general, vaccinations cause more harm than good” [5-point Likert scale, from absolutely disagree (1) to absolutely agree (5)].

#### 2.2.4 Personal acquaintances with cases of COVID-19

The items from the WHO model questionnaire were further developed to differentiate between COVID-19 cases of different severity (World Health Organization, 2020b). Participants were asked if they know personally an individual who has been ill with COVID-19 with one of three outcomes: Mild cases: “COVID-19 was officially diagnosed, and the person had no complications”; Hospitalization cases: “COVID-19 was officially diagnosed, and the person was hospitalized”; Fatal cases: “The person died from complications associated with COVID-19”. The respondents answered “yes” or “no” for each case. There was an option to skip the question.

#### 2.2.5 Subjective norms toward social distancing as COVID-19 preventive behavior

To measure subjective norms regarding social distancing, two items were created based on Ajzen's TPB Questionnaire Construction (Fishbein and Ajzen, 2010): “Most people who are important to me expect me to try to stay at home and keep distance from other people during the coronavirus epidemic” (injunctive norm), and “Most university students follow social distancing measures (try not to leave the house and keep their distance from other people)” (descriptive norm). Possible responses were rated on a 5-point Likert scale from “absolutely no” (1) to “absolutely yes” (5).

#### 2.2.6 Mental health symptomology

An adopted Russian-language version of the GAD-7 questionnaire (Generalized Anxiety Disorder-7, Spitzer et al., 2006) was used to assess participants' anxiety symptoms over the past 2 weeks on a 4-point Likert scale, ranging from 0 (never) to 3 (nearly every day). The total GAD-7 score ranged from 0 to 21, with higher scores indicating more severe functional impairments as a result of anxiety. Scores above ≥10 are considered to be in the clinical range (Kroenke et al., 2007).

An adopted Russian-language version of the PHQ-9 (the depression module of the Patient Health Questionnaire, Kroenke et al., 2001) was used to assess participants' depressive symptoms. The severity of depressive symptoms over the past 2 weeks was measured on a 4-point Likert scale ranging from never (0) to nearly every day (3). The total PHQ-9 score ranges from 0 to 27, cut-off points between 8 and 11, and higher scores indicate more severe depressive symptomatology (Manea et al., 2012).

The following one-item measure was included to describe the self-evaluation of mental health by students (Currie et al., 2014): “Do you think that your mental health is…?” with possible responses on a 4-point Likert scale: “poor” (4), “satisfactory” (3), “good” (2), and “excellent” (1).

#### 2.2.7 Psychosomatic complaints

According to WHO recommendations (World Health Organization Regional Office for Europe, 2016), a subjective representation of somatic health was assessed using the HBSC-SCL subscale. Students were asked how often they had experienced the following symptoms in the last 6 months: headache; stomach ache; feeling low, irritable or bad tempered; feeling nervous; difficulties in getting to sleep; and feeling dizzy. In the study, a question was added regarding pain in other parts of the body. Response options for each symptom ranged from (almost) every day (5) to rarely or never (1). Two indicators were calculated (World Health Organization Regional Office for Europe, 2016): Psychosomatic Health scale—the sum of the values for six parameters (excluding sleep disturbances and pain in other body parts) (min = 6, max = 30) and Psychosomatic Health Index—belonging to the group of people with at least two symptoms (out of eight measured) experienced several times per week or more.

#### 2.2.8 Perceived social support

An adopted Russian-language version of the MSPSS questionnaire (The Multidimensional Scale of Perceived Social Support; Zimet et al., 1988) was used to assess social support. Three subscales, each addressing a different source of support: Family (α = 0.714), Friends (α = 0.846), and Significant Other (α = 0.761) with score ranges from 0 to 4 for each. The total score ranged from 0 to 12.

### 2.3 Using the dataset

The dataset was prepared by merging the first wave of survey dataset with the second wave. The resulting dataset is structured in long-format and includes 76 quantitative variables. The dataset is as a CSV file. The data are fully anonymized and cleaned.

### 2.4 Data analysis

The indicators reflecting personal experience and acquaintances with cases of COVID-19, COVID-19 preventive behaviors, the endorsement of COVID-related misconceptions and conspiracy ideas, norm and trust, mental health symptoms, stratified by two waves of the pandemic, were reported; the chi-square test (χ^2^) and Mann–Whitney *U*-test were used to compare the differences between groups.

## 3 Some analysis of the data

During the early stages of the pandemic, only 39.5% of students knew someone who had contracted COVID-19. In October 2020, significantly more students faced cases of COVID with any types of outcomes from mild to the fatal ones (87%). Thus, awareness about COVID-19 cases in personal networks increased (Table 1).

**Table 1:** Descriptive statistics of the key variables in the first and second waves, %.

	**Spring 2020**		**Autumn 2020**		**Total**			
	***N***	**%**	***N***	**%**	***N***	**%**	**Chi-square**	***P***
**Perception of current COVID-19 stage**								
**The most difficult period of the epidemic of Coronavirus**								
Is in the past	215	21.1	99	12.8	314	17.5	38,790	0.000
Is now	370	36.4	384	49.7	754	42.1		
Is ahead	312	30.7	204	26.4	516	28.8		
Don't want to answer	120	11.8	86	11.1	206	11.5		
**Awareness about COVID-19 cases in personal network**								
With mild outcome	292	28.7	620	80.2	912	50.9	481,141	0.000
Hospitalized	205	20.2	365	47.2	570	31.8	165,254	0.000
Fatal	79	7.8	217	28.1	296	16.5	153,128	0.000
With any types of outcomes	381	39.5	647	87.0	1,028	60.2	394,38	0.000
**Psychosomatic health index**								
At least two symptoms are present	594	58.4	488	63.1	1,082	60.4	4,099	0.043
**Depression (PHQ-9)**								
Clinical level of depression (cut-off point ≥11)	393	38.6	371	48.0	764	42.7	15,700	0.001
**Anxiety (GAD-7)**								
Clinical level of anxiety (cut-off point ≥10)	117	11.5	102	13.2	219	12.2	2.494	0.476

COVID-19 prevention practices also have changed (Table 2). As we can see, a significant decrease is observed in the following types of preventive behavior: washing hands, social distancing, social isolation, and wearing gloves. But the most common protective behavior practice among students, such as wearing masks, remained on the same level.

**Table 2:** Descriptive statistics of the key variables in the first and second waves, mean.

	**Spring 2020, *M***	**Autumn 2020, *M***	**Total, *M***	**Mann-Whitney *U*/** **Kolmogorov-Smirnov *Z***	***P***
**Psychosomatic health scale** Min 6, Max 30	15.8	16.7	16.2	361,420.5	0.003
**Depression (PHQ-9)** Min 0, Max 27	9.9	11.1	10.4	357,793	0.001
**Anxiety (GAD-7)** Min 0, Max 21	6.5	6.8	6.6	381,778	0.296
**Self-evaluation of mental health** 1 (excellent)…4 (poor)	2.5	2.8	2.6	2.907	0.000
**COVID-19** ***prevention practices*** **1 (never)…5 (always)**					
Mostly stayed at home	4.0	3.5	3.8	4.446	0.000
Keep social distancing	4.1	3.3	3.8	5.694	0.000
Wearing masks	4.4	4.4	4.4	0.766	0.601
Wearing gloves	3.4	2.5	3.0	6.254	0.000
Washing hands	4.2	3.8	4.0	3.585	0.000
**Subjective norms 1 (no)…5 (yes)**					
Injunctive norm	4.9	4.5	4.7	1.807	0.003
Descriptive norm	3.5	3.1	3.3	5.332	0.000
**Misconceptions 1 (** ***disagree*** **)…5 (** ***agree*** **)**					
“There is no difference between Coronavirus and ordinary influenza”	2.6	2.3	2.4	2.997	0.000
“In order to avoid infection, it is enough to strengthen the immune system, for example, by using folk remedies and temper”	2.2	1.9	2.1	3.684	0.000
“Coronavirus is generally underestimated as a threat”	3.8	3.6	3.7	1.683	0.007
“In general, vaccines cause more harm than good”	2.2	2.4	2.3	2.421	0.000
**Trust 1 (** ***don't trust*** **)…5 (** ***trust*** **)**					
Trust in the official medicine recommendations	3.8	3.5	3.7	3.570	0.000
Trust in the government	2.6	2.4	2.5	1.433	0.033
**Perceived social support 1 (** ***min*** **)…4 (** ***max*** **)**					
Family	3.3	3.4	3.4	1.023	0.246
Friends	3.6	3.6	3.6	0.987	0.284
Significant other	3.6	3.6	3.6	0.388	0.998

Besides subjective normative beliefs regarding protective behavior are becoming more negative (Table 2). In the second wave students, on average, were less likely to believe that most important people expect them to try to stay at home and keep distance from other people during the coronavirus epidemic (injunctive norm), and that the majority of university students adhere to social distancing measures (descriptive norm).

At the same time, the social perceptions about the coronavirus changed. All measured COVID-related misconceptions and conspiracy ideas significantly reduced among students (Table 2). The notable exception is the trust in vaccination—there was an increase in the number of students who believe vaccination does more harm than good.

University students' trust in social institutions (both in official medicine and the government) in regard to COVID-19 decreased (Table 2).

A number of indicators of university students' mental health deteriorated, except for anxiety (Tables 1, 2). Mean mental health self-evaluations decreased significantly from good to satisfactory. During the second wave of survey, we observed an increase in clinically pronounced depressive symptoms (PHQ-9). But according to the GAD-7 there is no significant change in anxiety over time.

During both waves of survey psychosomatic complaints increased significantly: (both Psychosomatic Health scale and Psychosomatic Health Index, Tables 1, 2). During both waves of the pandemic, the following symptoms were prominent: irritability, irascibility, and anxiety.

Our study confirmed a negative dynamic in most analyzed indicators during an early stage of the pandemic (from May to October 2020) including a decrease in preventive measures and trust to officials, introducing of less favorable social norms regarding preventive behavior as well as increase of mental health symptomology.

The proposed database is convenient for studying the dynamics and interactions COVID-related behaviors and its factors. This dataset contains a large number of psychological variables and is one of few employed repeated cross-sectional designs.

## 4 Limitations

The sample consists primarily of females: the overall sample structure could limit the generalizability of reported findings.

Behavioral variables were self-reported and could possibly be prone to number of associated biases. However, those risks were mitigated by the anonymization applied in the study, as well as the fact that the survey questions in regard to behavior covered a 1-week period that is easy to remember.

## Data availability statement

The datasets presented in this study can be found in online repositories. The names of the repository/repositories and accession number(s) can be found below: https://osf.io/y5nf6/.

## Ethics statement

The studies involving humans were approved by Ethics Committee of University in Saint-Petersburg (IRB00011060 University of Russia IRB#1, Record #19). The studies were conducted in accordance with the local legislation and institutional requirements. Written informed consent for participation in this study was provided by all the participants.

## Author contributions

NA: Conceptualization, Methodology, Writing – original draft, Writing – review & editing. KE: Conceptualization, Methodology, Writing – review & editing. NU: Formal analysis, Writing – review & editing. LT: Investigation, Project administration, Writing – review & editing.
