# Supplementary material for: Dynamics of university students' experiences with COVID-19 in the first year of pandemic in Russia: dataset
Source: Front Psychol. 2023 Nov 15;14:1286036. doi: 10.3389/fpsyg.2023.1286036 (PMC10685103; doi:10.3389/fpsyg.2023.1286036)
Supplement: Supplementary file 1 [file Data_Sheet_1.docx]

***Supplementary Material***

**The codebook**

**Instructions for matching the data**

The two databases are presented separately.

The dataset_vertical_1790 combined from the both two waves of data collection consists of 1790 observation: 1017 in the first wave and 773 in the second. The variable “V1_waves” indicate the wave of the data collection for the particular case.

The dataset_horisontal_197 is longitudinal dataset with 197 unique respondents participated in the both waves of data collection. It provides opportunity to analyse within individual changes (longitudinal study). The respondent has the same ID and the time of the data collection (wave) is indicated in the label of the variables (for example, V2_t1 (for first wave of data collection) and V2_t2 (for second wave)).

| **Key domains** | **Item** | **Name in the database** | **Questions with scales** |
| --- | --- | --- | --- |
| **Waves** | V1 | waves | Waves of the study:  1 - May-June 2020  2- September-November 2020 |
| **Personal information** | ID | respondent ID | Respondent ID |
|  | V2 | course | Please indicate the course you are currently studying on  1 – 1st bachelor course  2 – 2nd bachelor course  3 – 3rd bachelor course  4 – 4th bachelor course  5 – 5th bachelor course  6 – 6th bachelor course  7 – 1st master course  8 – 2nd master course  9 - postgraduate study |
|  | V3 | study_format | What is your current form of education?  1 – full-time  2 - part-time |
|  | V4 | payment | You study  1 – for a fee  2 – free of charge |
|  | V5 | gender | Your gender  1 – Male  2 – Female |
|  | V6 | age | Age |
|  | V7 | location | What is your current location?  1 – city St. Petersburg  2 - Leningrad Region  3 – other region of Russian Federation  4 - outside the Russian Federation  5 – other  88 – don’t want to answer |
|  | V8 | type of accommodation | What type of accommodation do you live in?  1 – separate apartment  2 - rented apartment  3 – room in a communal apartment  4 – private house/part of house  5 – dormitory  6 - other  88 – don’t want to answer |
| **COVID-19 preventive behaviors** | V9 |  | To what extent do the following statements describe your behavior OVER THE PAST WEEK?  1 (never) 2 3 4 5 (always) |
|  | V9.1 | stay_home | I predominantly stayed home |
|  | V9.2 | keep_distance | I kept a distance of 1.5 meters or more from other people in public places |
|  | V9.3 | mask_wearing | I wore a mask in public places |
|  | V9.4 | use_gloves | I used gloves, napkins in order not to touch objects and surfaces in public places with bare hands |
|  | V9.5 | wash_hands | I washed my hands more often and more thoroughly than before the Coronavirus epidemic |
| **Respondents’ endorsement of COVID-related misconceptions and conspiracy ideas** | V10 |  | 1 (absolutely disagree) 2 3 4 5 (absolutely agree) |
|  | V10.1 | misconseption_1 | Coronavirus is not much different from the usual flu |
|  | V10.2 | misconseption_2 | To avoid infection, it is enough to strengthen the immune system, for example, use folk remedies |
|  | V10.3 | misconseption_3 | Coronavirus is generally underestimated as a threat (REV) |
|  | V10.4 | misconseption_4 | In general, vaccinations cause more harm than good |
| **Perception of current COVID-19 stage** | V11 | perception_covid_stage | In your opinion, the most difficult period of the development of the epidemic of the coronavirus infection in the city / locality where you are now  1 – already passed  2 – is now  3 – will be in the future  88 - don’t want to answer |
| **Personal acquaintances with cases of COVID-19** | V12.1 | mild_cases | Do you personally know someone who was officially diagnosed with COVID-19 and the person had no complications?  0 - no, don’t know  1 – yes, know  88 – difficult to answer |
|  | V12.2 | hospitalization_cases | Do you know someone who was diagnosed with COVID-19 (a new coronavirus infection) and was admitted to the hospital with complications?  0- no, don’t know  1 – yes, know  88 – difficult to answer |
|  | V12.3 | fatal_cases | Do you know someone who was diagnosed with COVID-19 (a new coronavirus infection) and died as a result of complications?  0- no, don’t know  1 – yes, know  88 – difficult to answer |
| **Subjective norms towards social distancing as** **COVID-19 preventive behavior** | V13.1 | injunctive norm | Most people who are important to me expect me to me try to stay at home and keep distance from other people during the coronavirus epidemic  1 (absolutely no) 2 3 4 5 (absolutely yes) |
|  | V13.2 | descriptive norm | Most university students follow social distancing measures (try not to leave the house and keep their distance from other people).  1 (absolutely no) 2 3 4 5 (absolutely yes) |
| **Trust in official institutions** | V14.1 | trust_medical | How much do you trust the OFFICIAL MEDICAL RECOMMENDATIONS regarding the prevention of coronavirus?  1 – absolutely don’t trust  2 – rather don’t trust  3 – difficult to say  4 – rather trust  5 – absolutely trust  88 – don't want to answer |
|  | V14.2 | trust_government | How much do you trust the Russian government to take care of its citizens in an epidemic situation?  1 – absolutely don’t trust  2 – rather don’t trust  3 – difficult to say  4 – rather trust  5 – absolutely trust  88 – don't want to answer |
| **Psychosomatic complaints** | V15 | Subjective representation of psychosomatic health from HBSC | During last six months how often did you feel…  1 – rarely or never  2 – almost each month  3 – almost each week  4 - more than once a week  5 – almost every day |
|  | V15.1 | HBSC _headache | Headache |
|  | V15.2 | HBSC _stomachache | stomach ache |
|  | V15.3 | HBSC _other_ache | pain in other body parts |
|  | V15.4 | HBSC _feeling_low | feeling low |
|  | V15.5 | HBSC _irritability | irritable or bad tempered |
|  | V15.6 | HBSC _ nervous | feeling nervous |
|  | V15.7 | HBSC _bad_sleep | difficulties in getting to sleep |
|  | V15.8 | HBSC _dizzy | feeling dizzy |
|  | V16 | HBSC_scale | Psychosomatic Health scale -  the sum of the values for six parameters (excluding sleep disturbances and pain in other body parts) (min=6, max=30) |
|  | V17 | HBSC_index | Psychosomatic Health Index - belonging to the group of people with at least two symptoms (out of eight measured) experienced several times per week or more. |
| **Mental health (self-evaluation)** | V18 | mental_health | Do you think that your mental health is…?  1 – excellent  2 - good  3 - satisfactory  4 - poor  88 – don’t want to answer |
| **Depression scale from PHQ (PHQ-9).** | V19 –V21 | Depression scale from PHQ. | Nine items, each of which is scored 0 to 3, providing a 0 to 27 severity score.  0 – not at all  1 – several days  2 – more than half the days  3 – nearly every day  Over the last 2 weeks, how often have you been bothered by any of the following… |
|  | V19.1 | PHQ9_a | Little interest or pleasure in doing things? |
|  | V19.2 | PHQ9_b | Feeling down, depressed, or hopeless? |
|  | V19.3 | PHQ9_c | Trouble falling or staying asleep, or sleeping too much? |
|  | V19.4 | PHQ9_d | Feeling tired or having little energy? |
|  | V19.5 | PHQ9_e | Poor appetite or overeating? |
|  | V19.6 | PHQ9_f | Feeling bad about yourself—or that you are a failure or have let yourself or your family down? |
|  | V19.7 | PHQ9_g | Trouble concentrating on things, such as reading the newspaper or watching television? |
|  | V19.8 | PHQ9_h | Moving or speaking so slowly that other people could have noticed? Or the opposite—being so fidgety or restless that you have been moving around a lot more than usual? |
|  | V19.9 | PHQ9_i | Thoughts that you would be better off dead or of hurting yourself in some way? |
|  | V20 | PHQ9_total_score | The total PHQ-9 score ranges from 0 to 27, cut-off points between 8 and 11, and higher scores indicate more severe depressive symptomatology |
|  | V21 | PHQ9_ difficult | How difficult have these problems made it for you to do your work, take care of things at home, or get along with other people?  1 – not difficult at all  2 – a little bit difficult  3 – very difficult  4 - extremely difficult  N/A – no answer |
| **Generalised Anxiety Disorder scale from GAD-7** | V22 – V23 | GAD7 | How often have you been bothered by the following problems over the past 2 weeks?    0 – not at all  1 – several days  2 – more than half the days  3 – nearly every day |
|  | V22.1 | GAD7_1 | Feeling nervous, anxious, or on edge |
|  | V22.2 | GAD7_2 | Not being able to stop or control worrying |
|  | V22.3 | GAD7_3 | Worrying too much about different things |
|  | V22.4 | GAD7_4 | Trouble relaxing |
|  | V22.5 | GAD7_5 | Being so restless that it's hard to sit still |
|  | V22.6 | GAD7_6 | Becoming easily annoyed or irritable |
|  | V22.7 | GAD7_7 | Feeling afraid as if something awful might happen |
|  | V23 | GAD7_total score | The total GAD-7 score ranged from 0 to 21 |
| **Perceived Social Support scale from MSPSS** | V24 – V25 | The Multidimensional Scale of Perceived Social Support | Please express your opinion (Yes/no)  0 - no  1 – yes  88 – difficult to say |
|  | V24.1 | MSPSS_1 | There is a special person who is around when I am in need. |
|  | V24.2 | MSPSS_2 | There is a special person with whom I can share my joys and sorrows |
|  | V24.3 | MSPSS_3 | My family really tries to help me |
|  | V24.4 | MSPSS_4 | I get the emotional help and support I need from my family |
|  | V24.5 | MSPSS_5 | I have a special person who is a real source of comfort to me |
|  | V24.6 | MSPSS_6 | My friends really try to help me |
|  | V24.7 | MSPSS_7 | I can count on my friends when things go wrong |
|  | V24.8 | MSPSS_8 | I can talk about my problems with my family |
|  | V24.9 | MSPSS_9 | I have friends with whom I can share my joys and sorrows |
|  | V24.10 | MSPSS_10 | There is someone special in my life who cares about my feelings |
|  | V24.11 | MSPSS_22 | My family is willing to help me make decisions |
|  | V24.12 | MSPSS_12 | I can talk about my problems with my friends |
|  | V25.1 | MSPSS_Family | Family Subscale: Sum across items 3, 4, 8, & 11, then divide by 4. |
|  | V25.2 | MSPSS_Friends | Friends Subscale: Sum across items 6, 7, 9, & 12, then divide by 4. |
|  | V25.3 | MSPSS_Others | Significant Other Subscale: Sum across items 1, 2, 5, & 10, then divide by 4. |
|  |  |  | Total Scale: Sum across all 12 items, then divide by 12. |
| **Academic performance** | V26.1 | academic_grade | Which grade best reflects your academic performance at the end of the last semester? (*In the University where dataset was collected ‘3-’ is a minimal pass grade (satisfactory) and ‘5’ is a maximum grade (excellent)).*  0 no grades  1 3-  2 3  3 3+  4 4-  5 4  6 4+  7 5-  8 5 |
|  | V26.2 | academic_debt | Do you have any academic debt?  0 No  1 Yes  88 Don’t know |
| **Working status** | V27 | working | Are you currently working in parallel with your studies at any paid job?  0 - No, I don’t work.  1 - Yes, I do, and this work is related to the profession I'm pursuing.  2 - Yes, I do, but this work is not related to the profession I'm pursuing. |
| **The next three indicators are available only for the second round of data collection.** | | | |
| **Test for Coronavirus** | V28 | covid_test | Have you ever been tested for Coronavirus (PCR or antibody test)?  0 – no  1 – yes, the last test result was negative  2 – yes, the last test result was positive  N/A – no answer |
| **Coronavirus disease** | V29 | covid_disease | Have you had a novel coronavirus infection (COVID-19)?  0 – No  1 – It seems that I was sick, but I did not perform a Coronavirus test.  2 – I believe I was sick, but the Coronavirus test came back negative.  3 – Although I had Coronavirus, I did not suffer any complications as a result of the infection.  4 – I had Coronavirus and I have been hospitalized with complications. |
| **Intention to vaccinate against coronavirus** | V30 | Intention_vaccinate | Will you vaccinate yourself against COVID-19 when a vaccine has successfully passed all phases of clinical trials?  1 - Absolutely no  2 - Probably not  3 - Rather yes  4 - Absolutely yes  5 - I have a medical reason for not being vaccinated  88 - Difficult to answer |
